# Prolonged Episodes of Paroxysmal Exertion-Induced Dystonia in Glut1 Deficiency Syndrome

**DOI:** 10.1055/a-2883-4228

**Published:** 2026-06-18

**Authors:** Joerg Klepper, Lucia Kiesel, Eva Runkel

**Affiliations:** 1 Department of Pediatrics/Neuropediatrics 9207 Childrens’ Hospital Aschaffenburg-AlzenauAschaffenburg, BavariaGermany; 2 Department of General Surgery 9207 Hospital Aschaffenburg-AlzenauAschaffenburg, BYGermany

**Keywords:** movement disorders, Glut1 deficiency, Glut1DS, ketogenic diet, PED, paroxysmal exertion-induced dystonia

## Abstract

Glucose transporter type I (Glut1) deficiency syndrome (Glut1DS) is associated with paroxysmal exertion-induced dystonia (PED). Episodes are published as brief, lasting 20 to 30 minutes. Increasingly, Glut1DS patients report prolonged PED of more than 1 hour duration. In a cohort of 60 Glut1DS patients seen in our institution within a 4-year period, a total of 26/60 patients (43%) on ketogenic dietary therapy (KDT) experienced PED. In 18/26 patients (30%), episodes lasted less than 1 hour as previously described. In 8/26 patients (13%), prolonged episodes lasted more than 1 hour up to 1 day despite adequate dietary treatment. We conclude that PED is common in Glut1DS starting in late childhood despite adequate treatment with KDT. Prolonged PED in Glut1DS occurs in a subset of patients, also unresponsive to KDT, and represents a substantial burden to patients and families.

## Introduction

Glut1 deficiency syndrome (Glut1DS) presents clinically with epilepsy, developmental delay, and movement disorders. Movement disorders can be constant or paroxysmal. Paroxysmal events are described as paroxysmal exertion-induced dystonia (PED) as they are often triggered by exercise or emotional distress. 1 Patients develop sudden dystonia, generally affecting the legs, most often one leg. When patients experience multiple episodes, symptoms may alternate between the right and the left leg. Dystonia is followed by the loss of motor control and gait disturbances of variable degree. Generally, episodes last several minutes and often resolve with rest. 1 2 Episodes are distressing to patients and families as they often come unexpectedly and, in the majority of cases, do not respond to ketogenic dietary therapies (KDTs). Recently, patients described prolonged, yet unreported, PED of more than 1 hour duration, which prompted this single-center, retrospective study analyzing the prevalence and duration of PED (<1-hour duration) and prolonged PED (>1-hour duration).

## Methods

PED duration and potential correlations were investigated in an outpatient cohort of 60 Glut1DS patients. Glut1DS was confirmed by pathogenic *SLC2A1* variants and/or hypoglycorrhachia as described. 1 All patients were seen in 6- to 12-month intervals in the outpatient clinic of Aschaffenburg Childrens' Hospital from 2021 to 2025. All patients (26 males, 34 females, mean age 14 years, range 2–26 years) were started on KDT following the diagnosis (classical ketogenic diet or Modified Atkins Diet). Ketosis ranged from 0.5 to 6 mmol/L 3-hydroxy-butyrate, depending on age. Informed consent for data collection and analysis was obtained from patients and/or parents based on the Declaration of Helsinki, October 2024.

## Results

The mean age and age ranges for diagnosis, start of KDT, and occurrence of PED are shown in Table 1 .

**Table 1:** Age (years) at diagnosis, start of ketogenic dietary therapy, and start of PED in 60 Glut1 deficiency syndrome patients, in 26 patients with PED, including the 8 patients with prolonged PED

	Total cohort ( *n* = 60)	PED ( *n* = 26)	Prolonged PED ( *n* = 8)
Mean age at diagnosis of Glut1DS	4.4 (0.2–17.2)	5.1 (0.3–17.2)	5.6 (0.8–15)
Mean age at start of KDT	4.6 (0.2–17.2)	5.3 (0.2–17.2)	5.7 (1–15.6)
Mean age at PED onset		9.4 (2–16)	9.2 (3–13)

Abbreviation: Glut1DS, Glut1 deficiency syndrome; KDT, ketogenic dietary therapy; PED, paroxysmal exertion-induced dystonia.

In all patients, the mean diagnosis of Glut1DS was about 5 years of age, and KDT was established promptly. On average, PED developed 4 to 5 years later despite KDT. PED and prolonged PED data did not differ significantly.

**Fig. 1:**
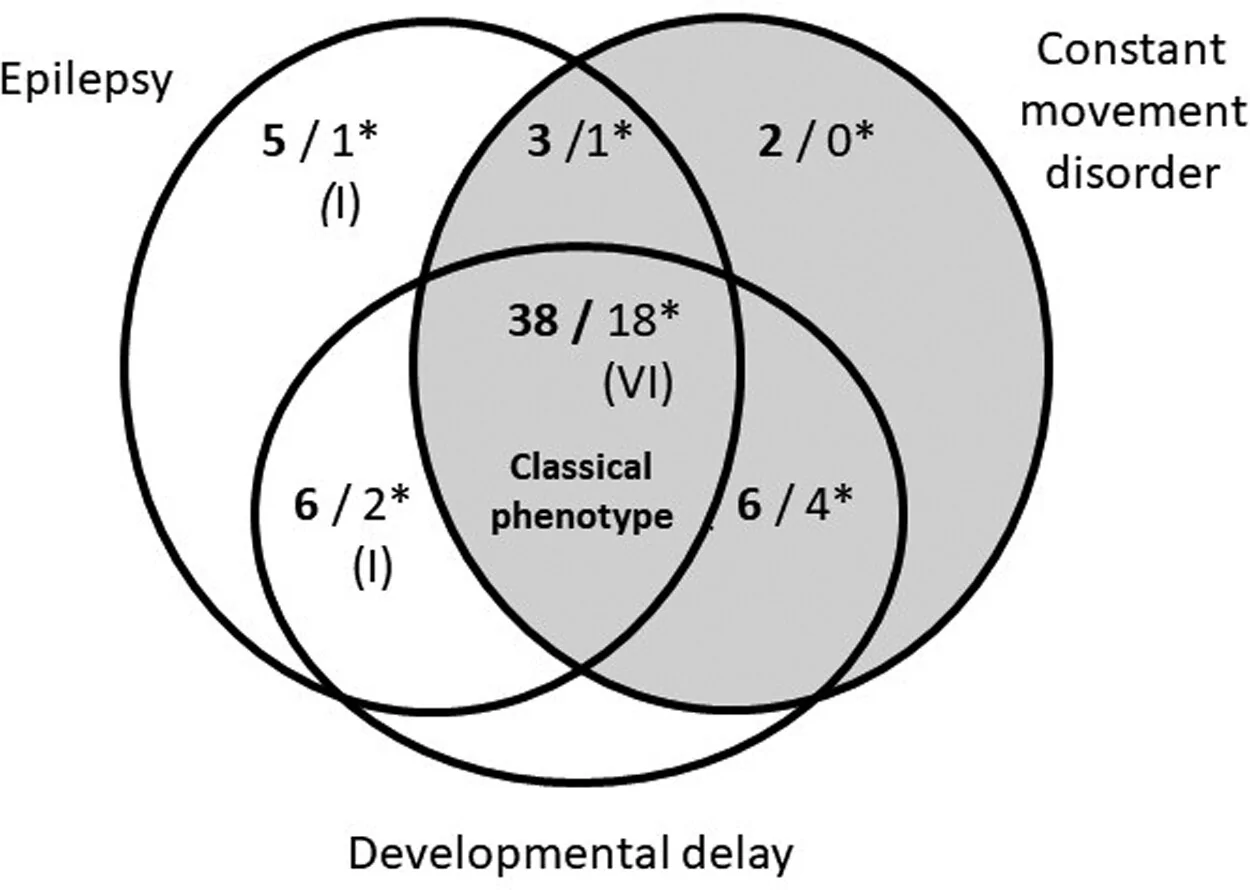
Glut1DS phenotype. Total number of patients ( *n* = 60) displaying isolated or combined features of epilepsy, constant movement disorder, and developmental delay (bold numbers). Patients with PED are indicated by (*). Within these groups, patients with prolonged PED ( *n* = 8) are shown in brackets and roman numerals. Glut1DS, Glut1 deficiency syndrome; PED, paroxysmal exertion-induced dystonia.

The Glut1DS phenotype in our cohort ( *n* = 60), including patients with PED ( *n* = 26), is shown in Fig. 1 .

The predominant phenotype, present in 38/60 patients, was classical Glut1DS, combining epilepsy, a constant movement disorder, and developmental delay. PED was present in 26/60 patients (43%). In 18/60 patients (30%), PED episodes lasted less than 1 hour as described previously. 2 Eight out of 60 patients (13%) developed prolonged PED episodes that lasted longer than 1 hour. The occurrence of PED (shown as number*) and prolonged PED (roman numerals) within individual phenotypes are depicted in Fig. 1 .

Prolonged PED duration varied from 1 to 2 hours (4/8 patients), 5 to 6 hours (1/8 patients), and up to 24 hours or longer (3/8 patients). In these patients, gender was evenly distributed (4/8 females, 4/8 males).

One patient described the occurrence of prolonged PED as associated with dietary slip-ups (chocolate on KDT), resulting in high ketones and high glucose in parallel. Acetazolamide was tried in 3/8 patients with prolonged PED <1 hour as described. 3 Two patients reported a positive effect, and the third patient discontinued acetazolamide for adverse effects. Levodopa (L-Dopa) or triheptanoin for PED were not tried in our cohort.

## Discussion

PED in Glut1DS is burdensome, common (43%), and often develops predominantly in late childhood (mean age 9.2 years), unresponsive to KDT. Though the majority of patients experienced self-limiting PED episodes, lasting 20 to 30 minutes, our study confirmed that prolonged episodes occur. As episodes often are unpredictable, distressing, and mostly unresponsive to KDT, they represent a difficult-to-treat symptom of Glut1DS. Alternative treatments discussed include acetazolamide, L-Dopa, and triheptanoin. 3 4 5 Among these compounds, triheptanoin was not tried in any patients based on the results of a multicenter trial. 3 No patient received L-Dopa, but interestingly, acetazolamide effectively mitigated prolonged PED in 2/8 individuals. Gender, age at diagnosis, or KDT initiation did not correlate with regular or prolonged PED occurrence. PED is common in children, adolescents, and adults with Glut1DS as described. 6 7 The confirmation of prolonged PED is of clinical importance as impairments by these episodes are even more distressing for patients. More data are needed to develop effective treatments for PED in Glut1DS.

## Conclusion

We conclude that PED is a common clinical feature in pediatric Glut1DS (26/60 patients; 43%) and is mostly unresponsive to KDT. Episodes may be prolonged, lasting over 1 hour (8/60 patients; 13%). PED represents a substantial burden to patients and families, and the need for alternative treatments is compelling.
